# Low-Dose, Ionizing Radiation and Age-Related Changes in Skeletal Microarchitecture

**DOI:** 10.1155/2012/481983

**Published:** 2012-04-18

**Authors:** Joshua S. Alwood, Akhilesh Kumar, Luan H. Tran, Angela Wang, Charles L. Limoli, Ruth K. Globus

**Affiliations:** ^1^Bone and Signaling Laboratory, NASA Ames Research Center, Mail Stop 236-7, Moffett Field, CA 94035, USA; ^2^Department of Radiation Oncology, University of California Irvine, Irvine, CA 92697, USA

## Abstract

Osteoporosis can profoundly affect the aged as a consequence of progressive bone loss; high-dose ionizing radiation can cause similar changes, although less is known about lower doses (≤100 cGy). We hypothesized that exposure to relatively low doses of gamma radiation accelerates structural changes characteristic of skeletal aging. Mice (C57BL/6J-10 wk old, male) were irradiated (total body; 0-sham, 1, 10 or 100 cGy ^137^Cs) and tissues harvested on the day of irradiation, 1 or 4 months later. Microcomputed tomography was used to quantify microarchitecture of high turnover, cancellous bone. Irradiation at 100 cGy caused transient microarchitectural changes over one month that were only evident at longer times in controls (4 months). *Ex vivo* bone cell differentiation from the marrow was unaffected by gamma radiation. In conclusion, acute ionizing gamma irradiation at 100 cGy (but not at 1 cGy or 10 cGy) exacerbated microarchitectural changes normally found during progressive, postpubertal aging prior to the onset of age-related osteoporosis.

## 1. Introduction

Osteoporosis can lead to devastating health consequences and poses a substantial health care burden currently affecting an estimated 44 million Americans (NIH). In women, estrogen deficiency is primarily responsible for the rapid postmenopausal loss of bone mass and resulting structural fragility (type I osteoporosis) while a slower bone loss (type II or senile osteoporosis) affects both men and women late in life [1]. Bone structure begins to decline early [2]; peak bone mass is achieved in early adulthood and progressively declines in both high turnover, cancellous tissue and in the surrounding hard cortical shell. The compartments of bone affected by osteoporosis can include both the dense tissue that forms an outer structural shell (cortical bone) and the spongy tissue (cancellous bone) within the marrow cavity; the microarchitecture of cancellous bone is profoundly affected in osteoporosis and renders sites that are rich in cancellous tissue, such as the spine and proximal femur, prone to fracture. An age-related decline in cancellous tissue structure occurs in rodents over a matter of months, rather than years, as in humans. C57BL/6J mice provide a well-established animal model with relatively rapid postpubertal loss of cancellous tissue [3, 4], characteristic of that which precedes the development of senile, type II osteoporosis in humans.

Bone turnover is regulated by a balance between the activities of bone-forming osteoblasts and bone-resorbing osteoclasts. Osteoblasts progressively differentiate from progenitors and mesenchymal stem cells whereas osteoclasts are haematopoietic derivatives of the monocyte/macrophage lineage. Stem cells, progenitors, and precursors of both lineages reside within the bone marrow. Bone marrow cells cultured *ex vivo *retain behavioral characteristics of *in vivo* function; thus bone marrow-derived cells cultured under conditions tailored either to stimulate osteoblast differentiation (osteoblastogenesis) or osteoclast differentiation (osteoclastogenesis) are useful tools to dissect cellular and molecular bases of physiological challenges such as skeletal aging and exposure to radiation [5–8].

Ionizing radiation generates reactive oxygen/nitrogen species (ROS/RNS) both by the ionization of target molecules and by perturbations to the cellular redox state, also implicated in the development of osteoporosis [9–12]. The production of ROS/RNS causes oxidative damage to DNA, lipids, and protein influencing key cell functions, including proliferation, differentiation, and apoptosis. Low Linear Energy Transfer (LET) ionizing radiation (e.g., gamma rays, X-rays) at high cumulative doses (>200 cGy) can damage vascular supply (osteoradionecrosis), leading to loss of cortical and cancellous tissue and leading to skeletal fragility [13], while adverse effects of lower doses (100–200 cGy) appear to be confined to cancellous tissue in animal models [12, 14–16]. Much less is known about how low doses influence skeletal health.

Here we hypothesized that low-to-moderate doses of ionizing radiation accelerate postpubertal and progressive loss of microarchitectural integrity over time. Ionizing radiation at 100 cGy (but not at 1 cGy or 10 cGy) caused structural changes in cancellous tissue within one month that occurred over 4 months in controls, with no further decrements following 1 month. The capability of marrow-derived cell populations to differentiate into osteoblasts or osteoclasts was unaffected.

## 2. Materials and Methods

### 2.1. Experiment Design

A total of 96 C57BL/6J 10-week old male mice (The Jackson Laboratory, Bar Harbor, ME) were housed 2/cage for up to 4 months. This age and duration of the experiment were selected on the basis of results reported by others, showing that this encompasses a period of microarchitectural changes that are associated with osteopenia (reduced bone density) over time [3, 4]. Mice were provided food (LabDiet 5001, Purina Mills, Gray Summit, MO) and water *ad libitum*. After three days of cage acclimation, unanesthetized mice (4 per exposure) were irradiated (IR; JL Shepherd Mark I, ^137^Cs gamma ray source, San Gabriel, CA) with 1, 10, or 100 cGy at 0.96, 0.96, or 88 cGy/min (±10%), respectively, or were sham-irradiated (0 cGy-controls). The exposure field was 26.5 × 19.5 cm^2^. To expose samples evenly, mice were placed in container (10 cm radius), centered 18 cm from the source, and rotated at 6 rpm. Radiation dose and uniformity was calibrated in air by JL Shepherd & Associates using an X-ray Monitor (no. 2025, MDH Industries, Monrovia, CA) with a 0.18 cm^3^ probe (National Institutes of Standards and Technology traceable). Tissues were harvested on the day of IR (basal) or 1 or 4 months later (*n* = 8/group), with cell culture being performed at the latter two endpoints. As an indicator of health, total body mass was measured (E0D120 scale, Ohaus Corporation, Parsippany, NJ) at least once per week until tissue harvest. The Institutional Animal Care and Use Committee of NASA Ames Research Center approved all procedures.

### 2.2. Cancellous Microarchitecture

Skeletal microarchitecture broadly characterizes the 3D network of cancellous (or spongy) bone. Microcomputed tomography (vivaCT 40, SCANCO Medical AG, Brüttisellen, Switzerland) allows direct quantification of the microarchitecture of mineralized tissue (10.5 *μ*m voxel size) within the proximal tibia metaphysis [17]. Standard indices [18] were generated within the excised right tibia in a region 0.2 to 1.2 mm distal to the proximal growth plate via semiautonomous contouring of cross-sectional slices, as reported in detail previously [15]. The relative amount of bone to total volume (BV/TV, bone volume fraction) quantifies the amount of bone present. The thickness of the bony struts, the thickness (or spacing) of the marrow voids, and trabecular number characterize trabecular morphologys on average [19]. Connectivity density quantifies the number of trabecular struts per volume in the bony network [20]. The structure model index measures the ratio of rod-like (=3) to plate-like (=0) trabeculae within the tissue [19]. The fabric tensor quantifies the anisotropic orientation of the structure, relating to mechanical properties [21, 22], with three eigenvalues describing the axial lengths of the anisotropy ellipsoid and the degree of anisotropy equaling the ratio of the maximum to minimum eigenvalues.

### 2.3. Primary Bone Marrow Culture

Marrow cells from the hindlimbs (femora and tibiae) were grown as previously described [7]. In brief, cells were flushed from the bone marrow and plated in *α*-MEM supplemented with 15% fetal calf serum with osteoblastogenic supplements (ascorbate 50 *μ*g/mL, Sigma-Aldrich, St. Louis, MO) and *β*-glycerophosphate (10 mM, Sigma-Aldrich, St. Louis, MO). For osteoclastogenesis, cells were plated overnight in macrophage colony stimulating factor (MCSF, 30 ng/mL, R&D Systems Inc, Minneapolis, MN) and nonadherent cells collected, replated, and grown in *α*-MEM supplemented with 15% fetal calf serum with MCSF (30 ng/mL) and soluble receptor activator of nuclear factor *κ*B ligand (sRANKL, 60 ng/mL, Peprotech, Rocky Hill, NJ). Osteoclastogenesis was assessed 4-5 days after euthanasia by counting the number of multinucleated, tartrate resistant acid phosphatase-positive (TRAP+, Sigma Aldrich) cells in a defined area by microscopy (between 18 and 45 mm^2^) to count a minimum of 200 cells. Osteoblastogenesis was assessed one month after irradiation by quantifying the surface area covered by alizarin red-stained, mineralized nodules.

### 2.4. Statistics

 Statistical analyses were performed using JMP 8 (SAS Institute Inc, Cary, NC). To address aging, three groups of sham controls were collected at different time points for analysis. Least squares linear regressions were performed with time as regressor and the null hypothesis of the slope being zero to assess changes over time. The coefficient of determination (*R* ^2^) and the probability (*P*) of the slope being zero are reported, with *P* = 0.05 the threshold for rejecting the null hypothesis.

For each time point after-irradiation, comparisons were made between the sham-irradiated and irradiated mice. Analyses proceeded using one-factor ANOVA with the Tukey-Kramer Honestly Significant Difference post hoc test, and *P* = 0.05 considered the threshold for significance. All figures report mean ± standard deviation (SD).

## 3. Results and Discussion

### 3.1. Body Mass

**Figure 1:**
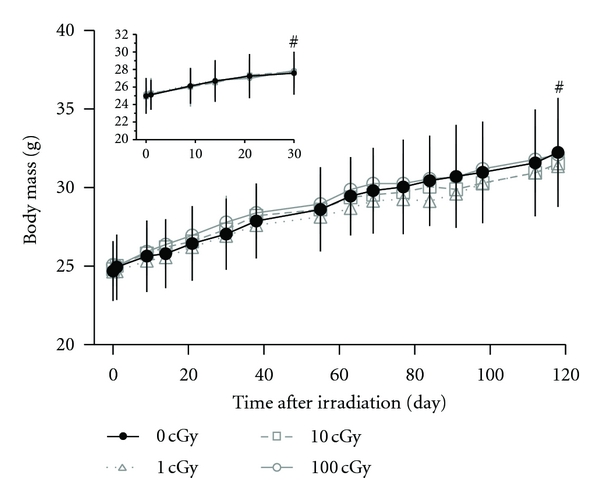
Mouse body mass as a function of age and radiation exposure. Experiment to determine whether a single dose of ^137^Cs gamma rays causes age-related changes in skeletal structure and bone cell differentiation. Male C57BL/6 mice (10 weeks of age, *n* = 8/group) were irradiated at 1, 10, and 100 cGy or were sham-irradiated (0 cGy) on day 0 and tissues harvested 1 month (inset) or 4 months after irradiation. Mean ± SD. ^#^Denotes *P* < 0.05 for changes with age (linear regression) for the respective cohorts (1 and 4 months after irradiation).

 Body weights were measured weekly during the experiment. Sham-irradiated mice gained 10% body mass from 10–14 wk of age (Figure 1. inset, *R* ^2^ = 0.96, *P* < 0.05 for slope) and 30% body mass from 10–27 wk of age (*R* ^2^ = 0.99, *P* < 0.05 for slope). Compared to the age-matched controls, irradiation at any dose did not affect the mass accrual (Figure 1). These data are consistent with previous studies that monitored radiation and aging effects [3, 4] and show that normal accrual of body mass, as a broad measure of health over time at this age, was unimpeded by exposure to gamma irradiation with these doses.

### 3.2. Cancellous Microarchitecture: Temporal Changes

**Figure 2:**
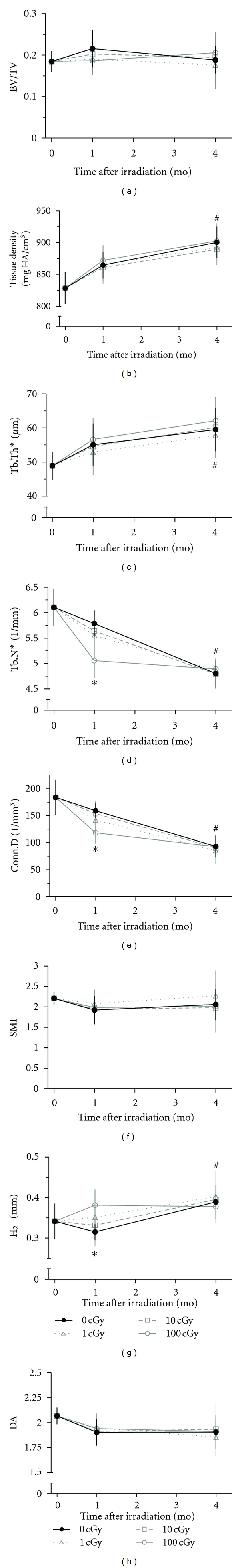
Cancellous microarchitecture of the proximal tibial metaphysis as a function of age and radiation exposure at the time of irradiation (basal, 0 months) and 1 and 4 months after irradiation. During aging, sham-irradiated controls show relative bone volume (BV/TV) remaining constant (a), increases in tissue density (b) and trabecular thickness (Tb.Th*) (c), and declines in trabecular number (Tb.N*) (d) and connectivity density (Conn.D) (e). The ratio of trabecular rods to plates, the structure model index (SMI), remains constant with age (f). The maximum eigenvalue (|H_2_|) of the fabric tensor increases with age (g), with the degree of anisotropy (h) remaining constant. At 1 month after irradiation, 100 cGy reduced Tb.N* by 20% (d), Conn.D by 36% (e) compared to age-matched controls. Irradiation at 100 cGy, but not at 1 cGy or 10 cGy, altered cancellous structure within 1 month in a direction and magnitude similar to 4 months of normal aging, with no further modulation between 1 and 4 months after irradiation. Mean ± SD. *Denotes *P* < 0.05 for changes with IR dose (ANOVA and Tukey-Kramer posthoc compared to age-matched sham controls). ^#^Denotes *P* < 0.05 for changes with age (linear regression).

Cancellous microarchitecture was evaluated using 3D microcomputed tomography. In sham-control mice, the cancellous bone volume fraction remained constant (Figure 2(a)), bone density increased, and most microarchitectural parameters declined as the mice aged over the 4-month period of this study. Trabecular number (−21%, *R* ^2^ = 0.79, *P* < 0.05) and connectivity density (−49%, *R* ^2^ = 0.75, *P* < 0.05) declined over 4 months (Figures 2(d) and 2(e)). Structure model index was unchanged within 4 months (Figure 2(f)). Each eigenvalue of the anisotropy tensor increased (all *P* < 0.05) within 4 months. The maximum eigenvalue increased 15%, *R* ^2^ = 0.30 (Figure 2(g)). The minimum eigenvalue increased 24%, *R* ^2^ = 0.49 (data not shown). The remaining eigenvalue increased 23%, *R* ^2^ = 0.59 (data not shown). The degree of anisotropy remained constant at 2.0 indicating a persistent longitudinal orientation of the structure (Figure 2(h)). In contrast to these indicators of increasing structural fragility with aging, trabecular thickness (+22%, *R* ^2^ = 0.35, *P* < 0.05) as in [3], and tissue density (+9%, *R* ^2^ = 0.59, *P* < 0.05) rose over 4 months (Figures 2(b) and 2(c)). 

We observed age-related changes in the cancellous microarchitecture of the tibia within the 4-month timeframe. In our study, declines in trabecular number and connectivity density ensued, while relative bone volume, rod-like morphology and degree of anisotropy remained constant. Concurrently, the cancellous tissue consolidated gains into thicker and denser, though fewer, trabecular struts. Trabeculae were spaced farther apart with age (data not shown) and may increase the propensity for strut buckling [23, 24]. These changes are consistent with normal aging of male C57BL/6 mice, including declines of trabecular number and connectivity density, which appeared to start sooner and fall more rapidly with aging than the bone volume fraction [3, 4]. In sum, these data suggest that age-related alterations of microarchitecture within the mouse tibia may precede overt declines in cancellous bone volume.

### 3.3. Cancellous Microarchitecture: Ionizing Radiation-Induced Changes

Radiation doses of 1 cGy or 10 cGy had no discernible effect on skeletal microarchitecture at either 1 or 4 months after-IR. In 100 cGy-IR mice, trabecular number and connectivity density declined (−20% and −36%, resp.; *P* < 0.05) and trabecular spacing and the maximum eigenvalues of the fabric tensor increased (+28% and +21%, resp.; *P* < 0.05) at one month after-IR compared to age-matched controls (Figure 2). There were no additional decrements in microarchitecture compared to age-matched controls between 1 and 4 months after-IR (Figure 2). These results indicate that 100 cGy irradiation caused rapid removal of trabecular, which resembled the normal microarchitectural changes expected to occur during 4 months of aging.

Irradiation with 100 cGy caused reductions in trabecular number and connectivity density and increases in the major axis of the anisotropy ellipsoid within 1 month—changes that take 4 months to appear in sham-irradiated controls. In previous studies that used older mice (16 weeks of age), 100 cGy caused bone loss in the tibia via reduced trabecular thickness, but not trabecular number, with the onset of osteopenia appearing 10 days after-irradiation [15]. The 16-week-old mice are past peak cancellous bone volume in the tibia, whereas the 10-week-old mice in this study have not yet begun to decline. Microarchitectural changes following irradiation observed in this study resembled the effects of a larger dose in older, male mice (16 weeks, 200 cGy) within the lumbar spine (a denser, plate-like cancellous tissue) [15] and in the tibia (unpublished results), where gamma irradiation at 200 cGy caused vertebral and tibial bone loss via trabecular number and connectivity density decrements appearing within 3 days and persisting at least until 30 days after-irradiation. This supports trabecular thinning preceding strut removal during bone resorption. Furthermore, in young female mice (9 weeks), osteopenia and trabecular number and connectivity density decrements persist in the tibia at 120 days after-irradiation [14]. Taken together, the data indicate that bone resorption is a time-dependent process contingent on initial bone volume and morphology (i.e., structure model index) and that skeletal outcome is a function of age.

In this study, microarchitectural differences between irradiated and age-matched controls were not evident 4 months after exposure to 100 cGy radiation. This result contrasts to the persistence of osteopenia in the tibia after low LET irradiation at higher doses (≥200 cGy X-ray or gamma rays) or with particulate irradiation at doses of 100 cGy (proton) [25] and 200 cGy (^56^Fe) [14] which present at 120 days after-irradiation in female mice (9 weeks). These findings highlight the importance of age at the time of irradiation; gender also may influence skeletal radioresponses and further study is needed in this area.

### 3.4. Bone Cell Differentiation Potential

**Figure 3:**
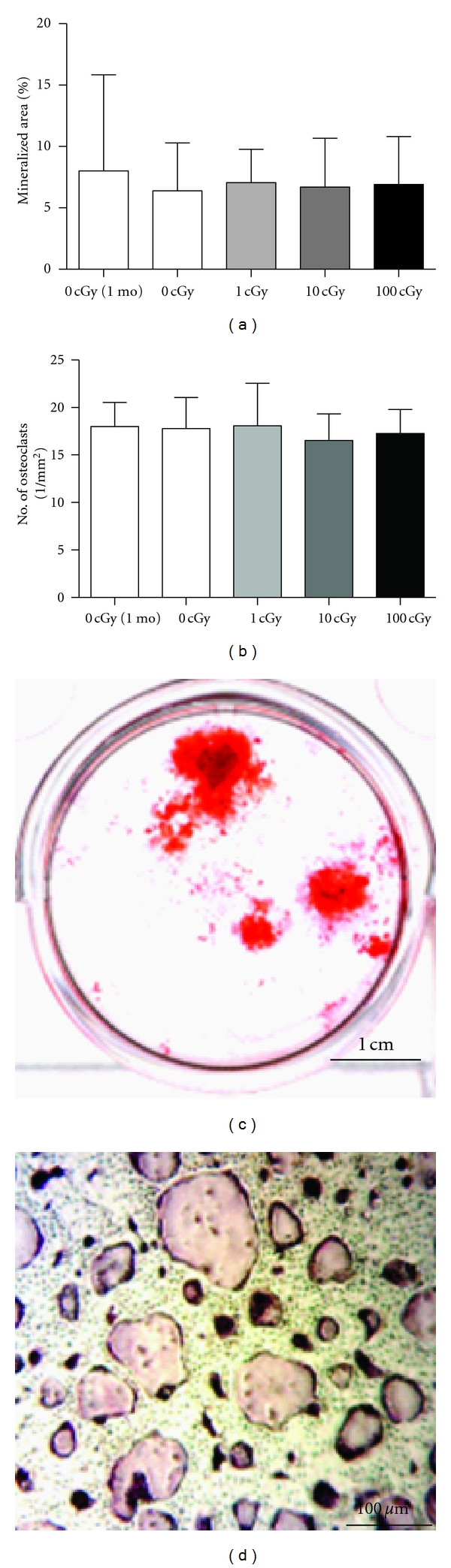
Bone-cell differentiation potential as a function of age and radiation exposure. Hindlimb bone marrow was flushed and cultured under osteoblastogenic or osteoclastogenic conditions to assess differentiation potential of the marrow. Between 1 and 4 months of aging (white columns), osteoblastogenesis (a), measured as the area of calcified matrix per well, and osteoclastogenesis (b), measured as number of TRAP-positive, multinuclear (≥3/cell) cells per area, remained constant in sham-control mice. Irradiation, at any dose (gray or black columns), did not affect the differentiation potential of bone cells in the marrow at 1 (not shown) or 4 months post-IR (a, b). Images of alizarin red-stained mineralized nodules (c) and TRAP-stained osteoclasts (d).

To assess differentiation of osteoblast lineage progenitors and precursors in the mesenchymal stem cell lineage, adherent cells from bone marrow were cultured in osteogenic medium up to one month and then stained for mineralized nodules (Figure 3). Irradiation did not affect the area covered by mineralized nodules (Figure 3(a)). These data suggest the osteoblast progeny of irradiated cells are not affected by a single, acute dose of low LET, ionizing radiation.

Bone marrow cells were cultured after tissue harvest in osteoclastogenic media and then osteoclast number counted. In sham-irradiated controls, between 1 and 4 months after-IR, the density of osteoclasts remained constant (Figure 3(b)) and indicates the number of precursors did not vary with age. Furthermore, irradiation at any dose did not affect the differentiation potential of osteoclasts compared to controls. Per animal, the ratio of nodule area fraction to osteoclast density was calculated as a measure of the balance between formation and resorption potential; this was unchanged in controls between 1 and 4 months and unchanged after irradiation at any dose (data not shown).

Thus, neither osteoblastogenesis nor osteoclastogenesis by bone marrow cells was affected by gamma irradiation, consistent with our previous findings. These results suggest the ability of marrow-derived progenitors to contribute to skeletal remodeling is preserved, despite exposure to ionizing radiation. Gamma rays or X-rays (200 cGy) can cause a rapid (within 3 days) increase in the numbers of osteoclasts lining the surfaces of trabeculae [15, 16], and a decrement in actively mineralizing surfaces, an index of the activity of mature, active osteoblasts [12]. Taken together, our results suggest that mature cells of the osteoblast and osteoclast lineage, rather than progenitors, are responsible for the near-term microarchitectural changes triggered by gamma radiation.

A limitation of this study design is that bones were analyzed at only two time points after exposure to radiation. Additional timepoints would be useful to further characterize the influence of radiation on age-related changes in skeletal health.

## 4. Conclusions

A moderate dose (100 cGy) of gamma radiation accelerated changes in the trabecular microarchitecture of postpubertal, growing (10 wk old) male C57BL/6 mice at a rate comparable to later skeletal aging in controls, whereas no effects were observed at even lower doses (1 or 10 cGy). Together with the finding that BV/TV did not differ over this time, these results suggest age-related microarchitectural changes precede bone loss. Bone marrow-derived osteoblast progenitors and osteoclast precursors did not appear to be affected by the low-to-moderate doses of low LET gamma radiation used here, suggesting stem and progenitor cells that reside in the marrow will retain the ability to contribute to skeletal remodeling during subsequent aging following the low-to-moderate doses employed. In contrast, exposure to high doses of gamma radiation [6] or to high LET irradiation such as ^56^Fe can inflict acute and persistent damage to osteoprogenitors ([7], unpublished observations). In conclusion, ionizing gamma irradiation at 100 cGy (but not at 1 cGy or 10 cGy) appeared to accelerate structural changes in the murine skeleton, expected also to occur during progressive, postpubertal aging in humans prior to the onset of type II osteoporosis.
